# Supplementary figures and images for: Muscle preflex response to perturbations in locomotion: In vitro experiments and simulations with realistic boundary conditions
Source: Front Bioeng Biotechnol. 2023 Apr 27;11:1150170. doi: 10.3389/fbioe.2023.1150170 (PMC10194126; doi:10.3389/fbioe.2023.1150170)

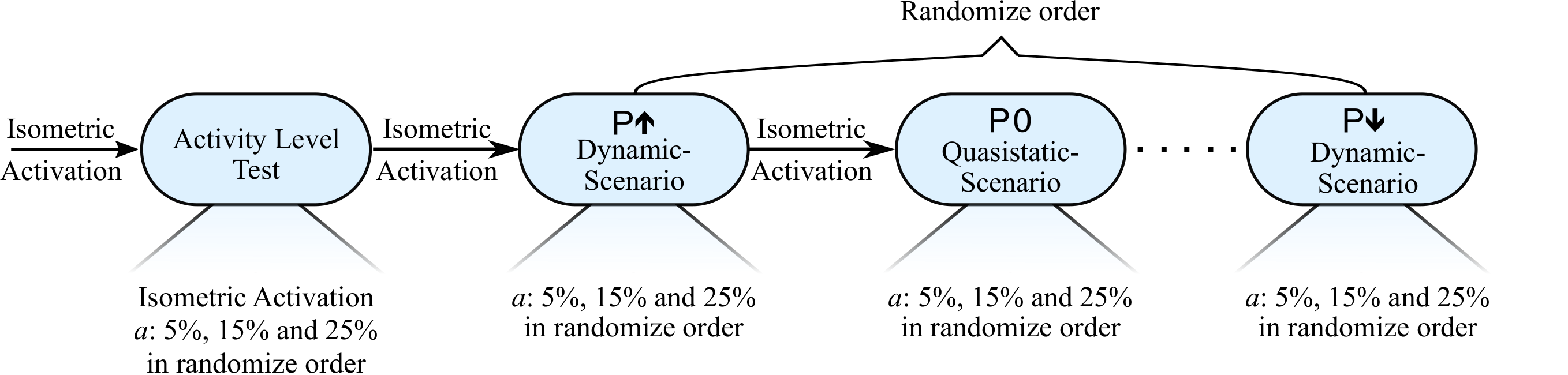

Supplement: Supplementary file 1 [file Image3.TIFF]

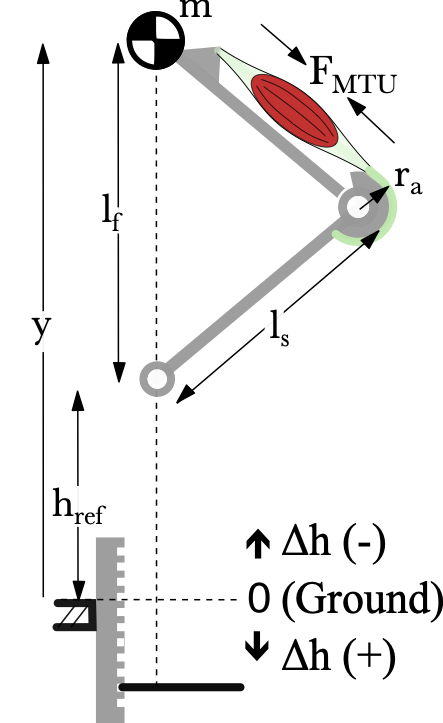

Supplement: Supplementary file 3 [file Image1.TIFF]

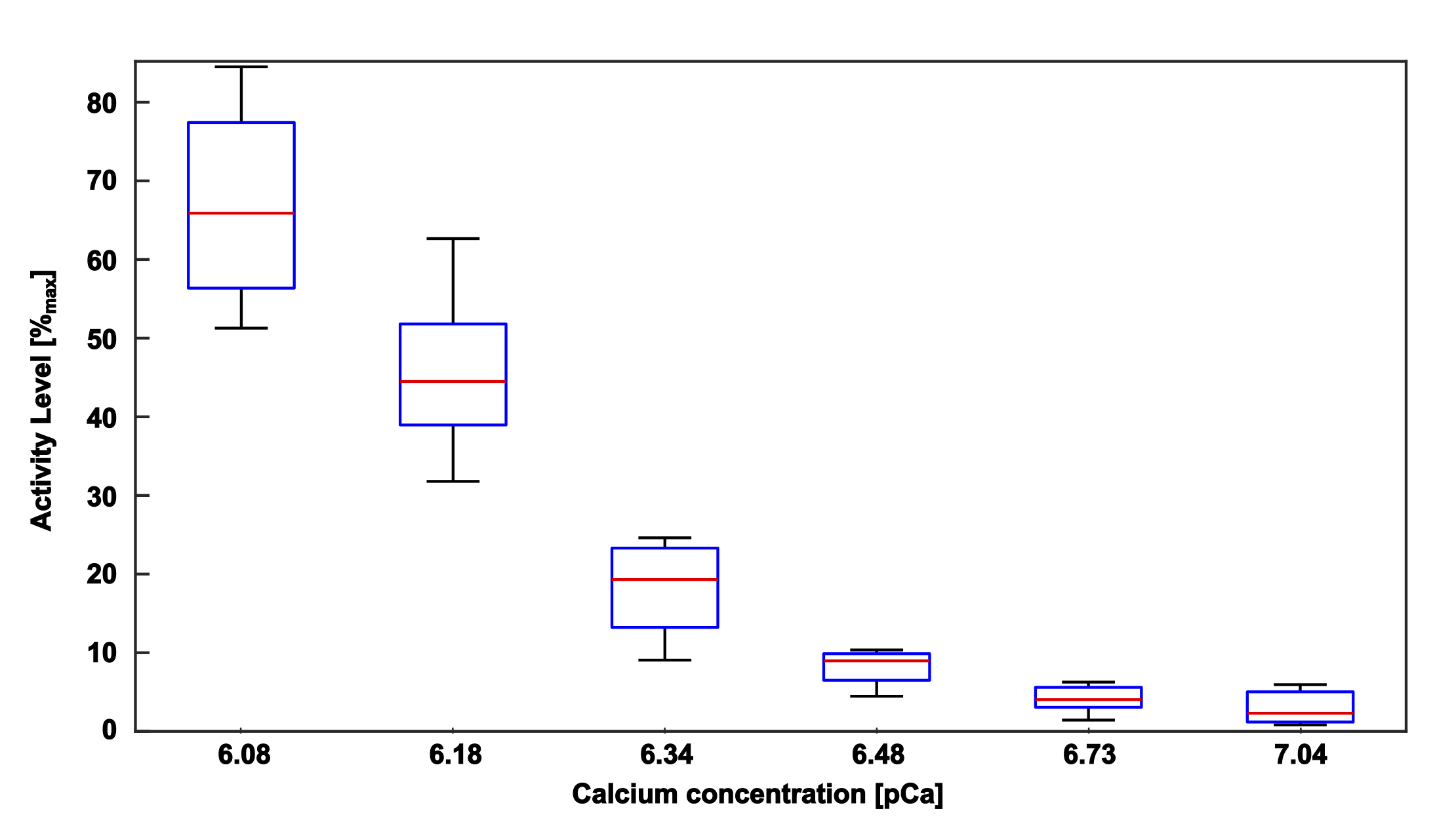

Supplement: Supplementary file 7 [file Image2.TIFF]
