# Supplementary material for: Muscle preflex response to perturbations in locomotion: In vitro experiments and simulations with realistic boundary conditions
Source: Front Bioeng Biotechnol. 2023 Apr 27;11:1150170. doi: 10.3389/fbioe.2023.1150170 (PMC10194126; doi:10.3389/fbioe.2023.1150170)
Supplement: Supplementary file 5 [file DataSheet1.pdf]

SUPPLEMENTARY MATERIAL

S.1 Hopping model details

The biomechanical model that we used for simulating vertical hopping was developed in previous studies (Geyer et al., 2003; Izzi et al., 2023). As shown in Fig. S1, it consists of a two-segment leg with mass lumped at the hip joint. The two segments have the same length $l_s = 0.5\,\mathrm{m}$, and the model mass is $m = 80\,\mathrm{kg}$. The model motion is constrained to the vertical direction and driven by a muscle-tendon unit, which applies an extending torque to the knee joint:

$$T = r_a\, F_{MTU}$$

Here, r_a is the lever arm at the knee joint; F_{MTU} is the force produced by the muscle-tendon unit according to the muscle architecture presented in (Haeufle et al., 2014) and activation dynamics in (Rockenfeller et al., 2015; Hatze, 1977). The muscle-tendon unit applies a force to the knee joint only during the stance phase, which is the phase of the hopping cycle with the leg foot in contact with the ground. During the flight phase (i.e., no ground-foot contact) the model geometry is fixed, with leg length $l_f = 0.99\,\mathrm{m}$. Given a reference hopping height (h_{ref}), step-up (-) and step-down (+) perturbations can be applied by altering the ground level of a quantity Δh. Tab. S1 gives a list of the model parameters used for this study.

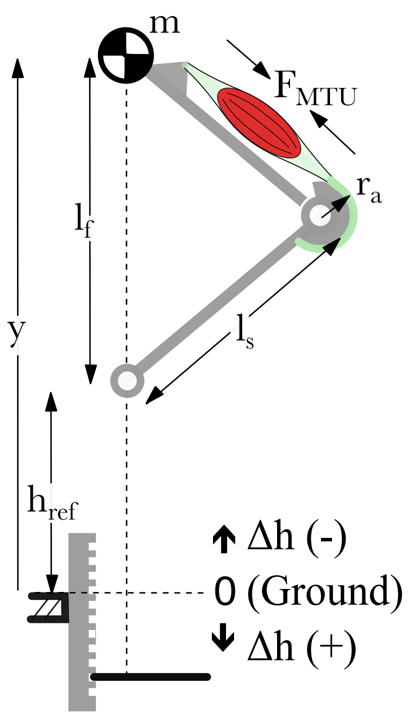

Figure S1. Biomechanical model used for simulating vertical hopping. Figure extracted from Figure 1**b** in Izzi et al. (2023).

Table S1. Parameters used in the leg-muscle model and activation dynamics (Hatze). Table derived from Table 1 and Table S1 in (Izzi et al., 2023), which are adaptations of Table 1 and Table 3 in (Geyer et al., 2003) and (Stollenmaier and Haeufle, 2019), respectively.

	Parameter	Unit	Value	Source	Description
General	g	m/s^2	9.81	(Geyer et al., 2003)	gravitational constant
	ℓ_f	m	0.99	(Geyer et al., 2003)	assumed flight leg length
	ℓ_s	m	0.5	(Geyer et al., 2003)	segment length
	m	kg	80	(Geyer et al., 2003)	body weight
	r_a	m	0.04	(Geyer et al., 2003)	knee joint lever arm
	h_{ref}	cm	9.27		reference hopping height producing periodic hopping
MTU	$l_{MTU,ref}$	m	0.5	(Geyer et al., 2003)	muscle-tendon unit's reference length, alias l_{ref} in (Geyer et al., 2003)
	F_max	kN	22	(Geyer et al., 2003)	maximum isometric force
CE	ℓ_opt	m	0.1	(Geyer et al., 2003)	optimum length contractile element
	ΔW^{des}	[ ]	0.45	similar to (Bayer et al., 2017); (Kistemaker et al., 2006)	width of the normalized bell curve in the descending branch, adapted to match observed force-length curves
	ΔW^{asc}	[ ]	0.4		width of the normalized bell curve in the ascending branch, adapted to match experimented isometric force-length curves
	$\nu_{CE,des}$	[ ]	1.5	(Mörl et al., 2012)	exponent for descending branch of force-length relation

...

Continued on next page

Table S1 – *Continued from previous page*

	Parameter	Unit	Value	Source	Description
	$\nu_{CE,asc}$	[ ]	3.8		exponent for ascending branch of force-length relation adapted to match experimented isometric force-length curves
	$A_{rel,0}$	[ ]	0.2	(Bayer et al., 2017)	parameter for contraction dynamics: maximum value of A_{rel}
	$B_{rel,0}$	1/s	2.0	(Bayer et al., 2017)	parameter for contraction dynamics: maximum value of B_{rel}
	$\mathcal{S}_{ecc}$	[ ]	2.0	(van Soest and Bobbert, 1993)	ratio of the derivatives of the force-velocity relation at the transition point ($v_{CE} = 0\,\mathrm{m/s}$)
	$\mathcal{F}_{ecc}$	[ ]	1.5	(van Soest and Bobbert, 1993)	factor by which the force can exceed F_{isom} for large eccentric velocities
PEE	$\mathcal{L}_{PEE,0}$	[ ]	0.95	(Bayer et al., 2017)	rest length of PEE normalized to optimal length of CE
	ν_{PEE}	[ ]	2.5	(Mörl et al., 2012)	exponent of F_{PEE}
	$\mathcal{F}_{PEE}$	[ ]	2.0	(Mörl et al., 2012)	force of PEE if l_{CE} is stretched to ΔW_{des}
SDE	D_{SDE}	[ ]	0.3	(Mörl et al., 2012)	dimensionless factor to scale $d_{SDE,max}$
	R_{SDE}	[ ]	0.01	(Mörl et al., 2012)	minimum value of d_{SDE} (at $F_{MTU} = 0\mathrm{N}$), normalized to $d_{SDE,max}$
SEE	$l_{SEE,0}$	m	0.4	(Geyer et al., 2003)	tendon's rest length, alias l_{rest} in (Geyer et al., 2003)
	$\Delta U_{SEE,nll}$	[ ]	0.0425	(Mörl et al., 2012)	relative stretch at non-linear linear transition
	$\Delta U_{SEE,l}$	[ ]	0.017	(Mörl et al., 2012)	relative additional stretch in the linear part providing a force increase of $\Delta F_{SEE,0}$
	$\Delta F_{SEE,0}$	N	0.4 F_{max}	(Bayer et al., 2017)	both force at the transition and force increase in the linear part

...

Continued on next page

Table S1 – *Continued from previous page*

	Parameter	Unit	Value	Source	Description
Hatze	m	1/s	11.3	(Kistemaker et al., 2006)	inverse of time constant for the activation dynamics
	c	mol/l	1.37e-4	(Kistemaker et al., 2006)	constant for the activation dynamics
	μ	l/mol	5.27e4	(Kistemaker et al., 2006)	constant for the activation dynamics
	k	[ ]	2.9	(Kistemaker et al., 2006)	constant for the activation dynamics
	q_0	[ ]	0.005	(Kistemaker et al., 2006)	resting active state for all activated muscle fibers
	ν	[ ]	3	(Kistemaker et al., 2006)	constant for the activation dynamics

S.2 Supplementary figures and tables

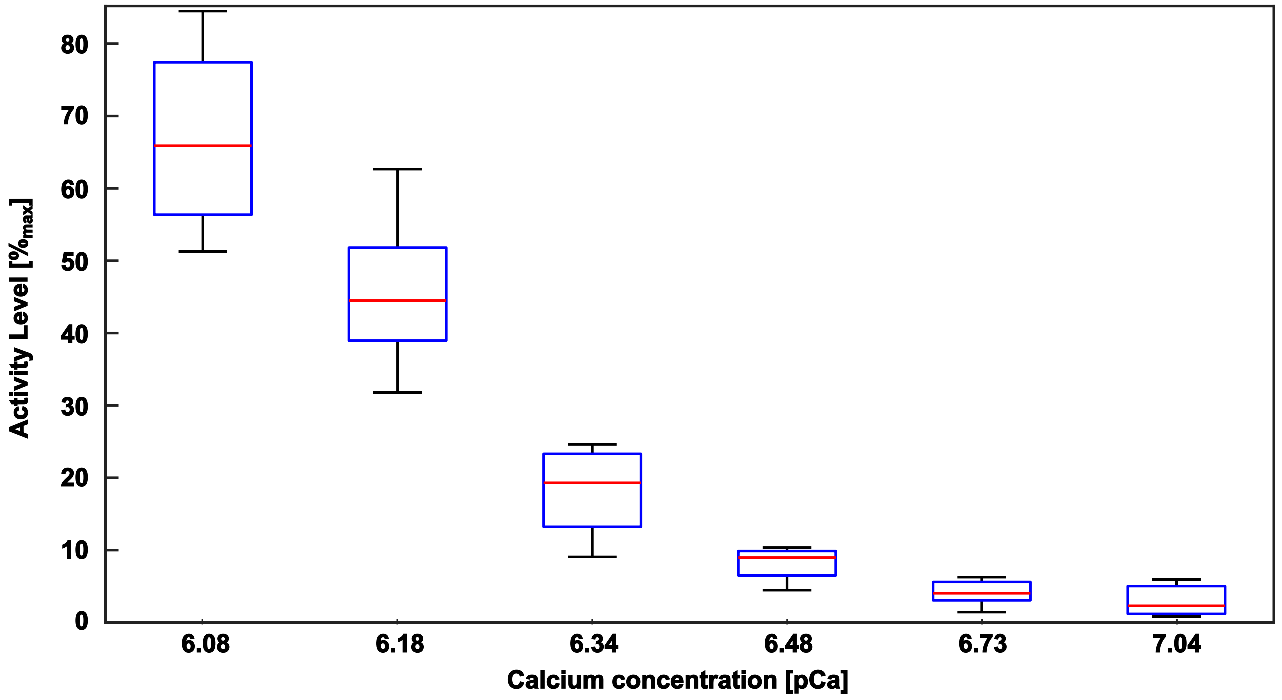

Figure S2. Measured activity level of skinned fibers (n=7) from the muscle used in the current study depending on the pCa of the experimental solution. Temperature while testing was 12 °C as in the perturbation experiment.

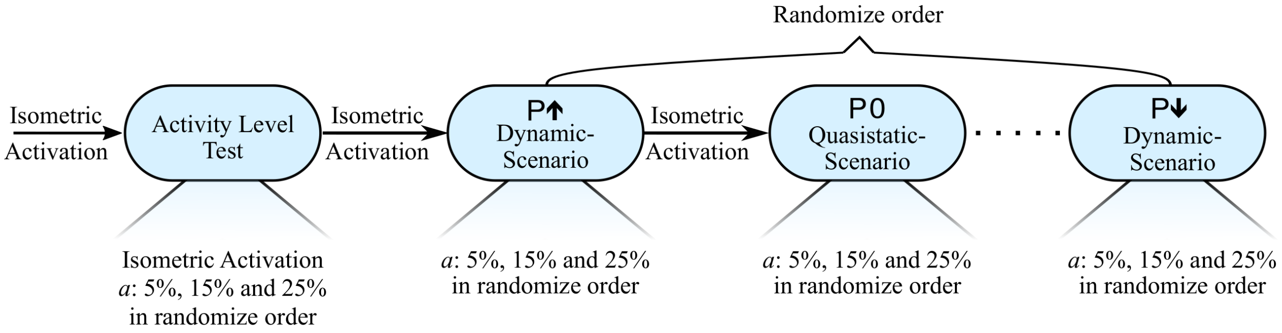

Figure S3. The Flow chart of an experimental day is shown here. First the activity level for every fiber was checked using the 6.73 , 6.34 and 6.3 pCa concentration solution to ensure that the experiment is matching the simulation condition. Afterwards the experimental blocks were conducted. One block contained all contractions (n=3) of a perturbation for one velocity-scenario. The order of the blocks were randomized on the day of the experiment. Between two blocks a reference contraction at optimal length and full activity was conducted to check for the degradation of the skinned fiber.

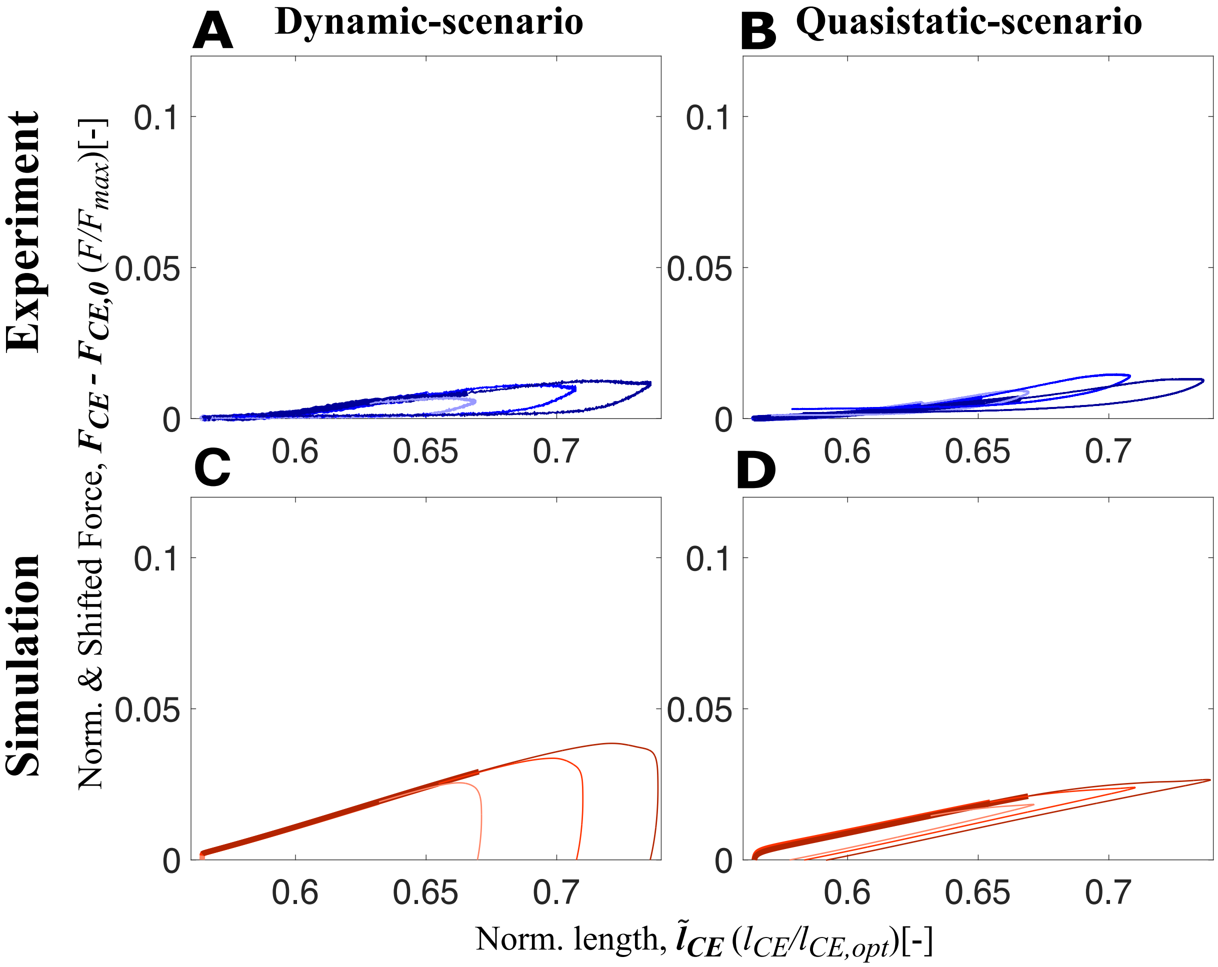

Figure S4. Shifted work loops for dynamic-scenario and quasistatic-scenario analysis step up(P↑), no (P0) and step down (P↓) perturbations for both experiments (**A-B**) and simulations (**C-D**) at 5 % activity level. The experimental data presented on **A** and **B** show the mean of all experimental trials. From touch-down to toe-off, all stretch-shortening cycle loops are plotted in the clockwise direction, and the thick and thin sections of the loops represent the preflex and remaining part of the stretch-shortening cycle, respectively.

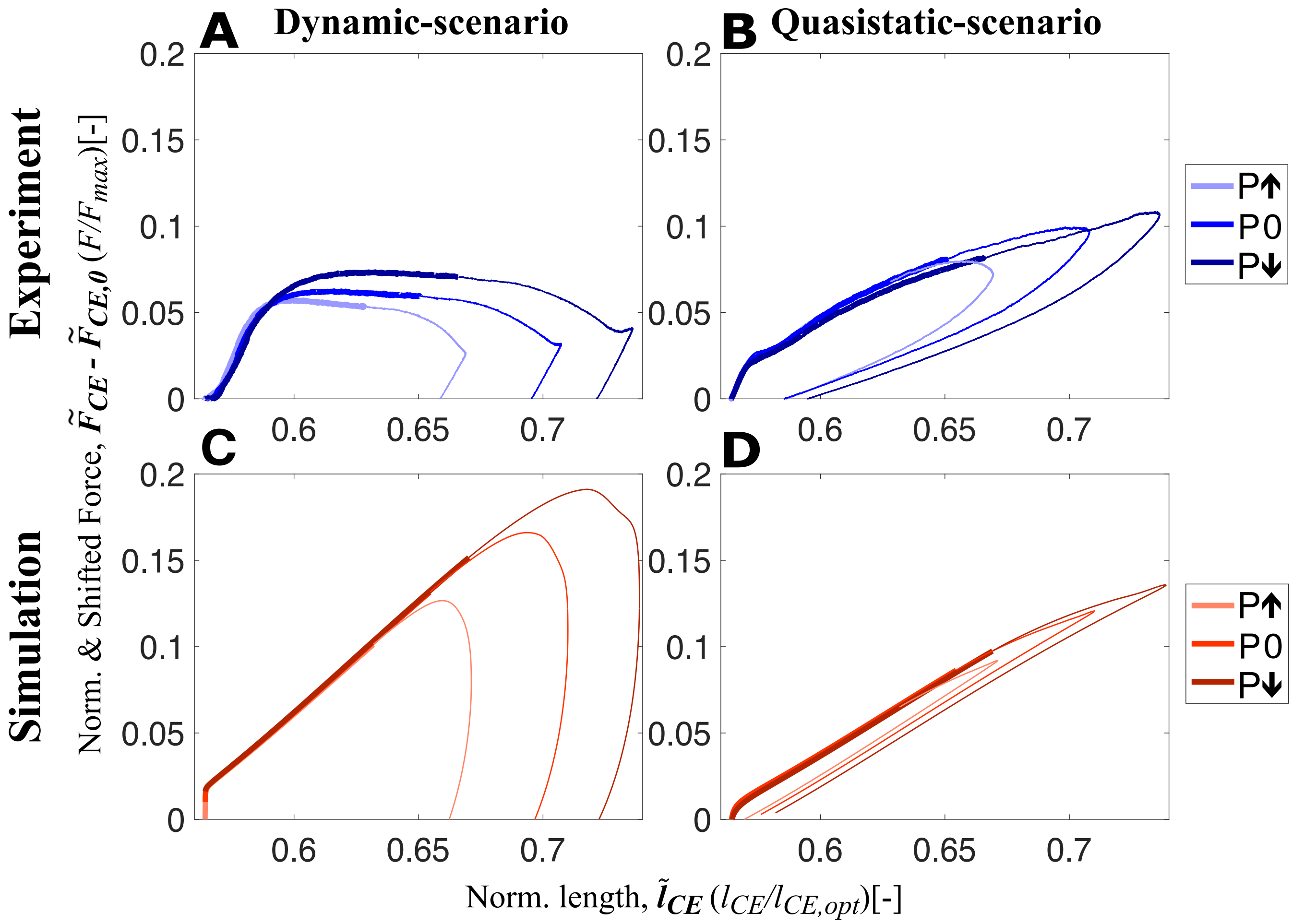

Figure S5. Shifted work loops for dynamic-scenario and quasistatic-scenario analysis step up(P↑), no (P0) and step down (P↓) perturbations for both experiments (**A-B**) and simulations (**C-D**) at 25 % activity level. The experimental data presented on **A** and **B** show the mean of all experimental trials. From touch-down to toe-off, all stretch-shortening cycle loops are plotted in the clockwise direction, and the thick and thin sections of the loops represent the preflex and remaining part of the stretch-shortening cycle, respectively.

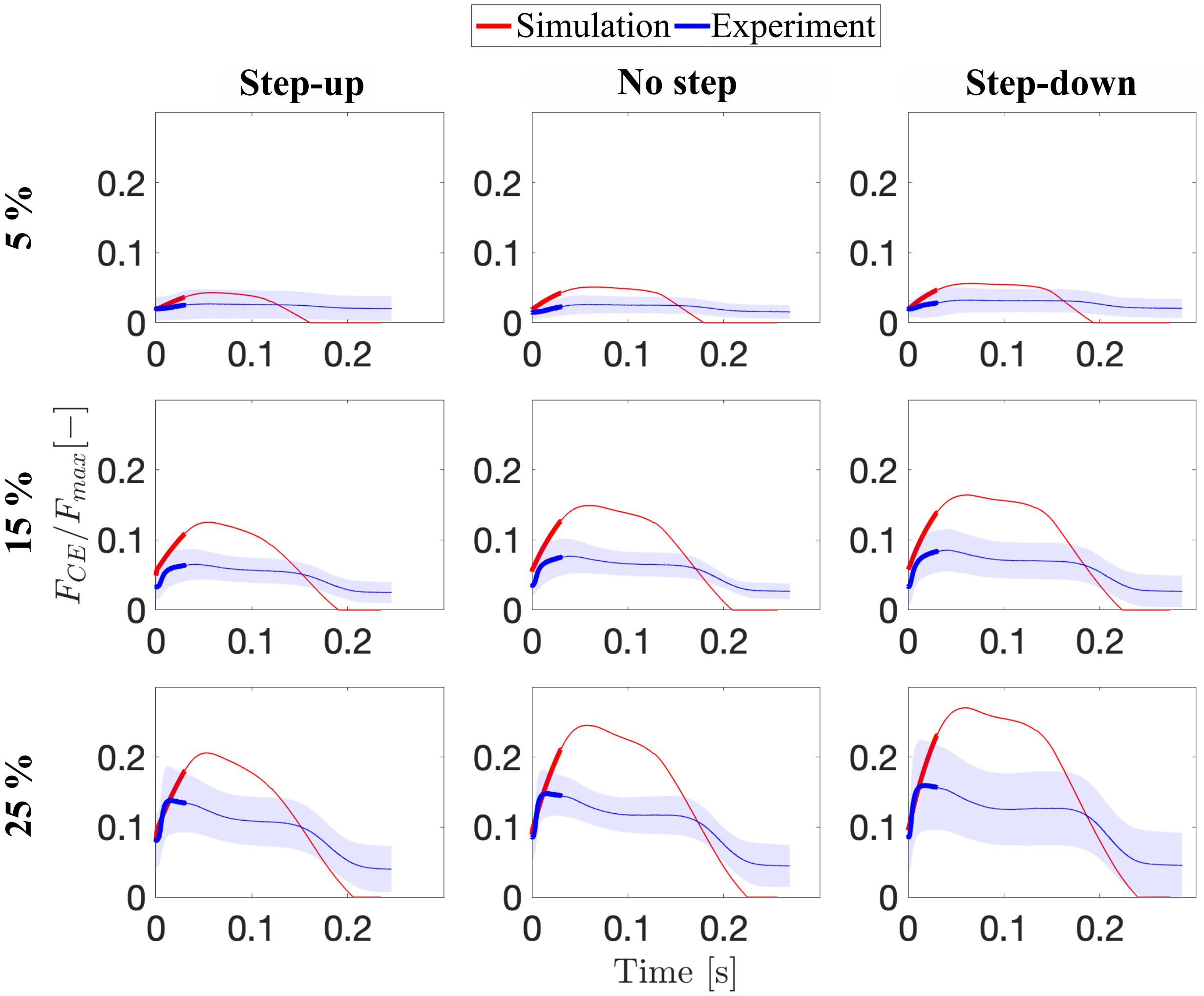

Figure S6. Force generated in dynamic-scenario by both muscle fibers and the Hill-type muscle model during one hopping cycle — from touch-down to toe-off — are presented for all perturbation and activity levels. The thick and thin sections of the loops represent the preflex and remaining part of the stretch-shortening cycle, respectively. the blue line and shaded area represent the mean of all experimental trials and the standard deviation, respectively.

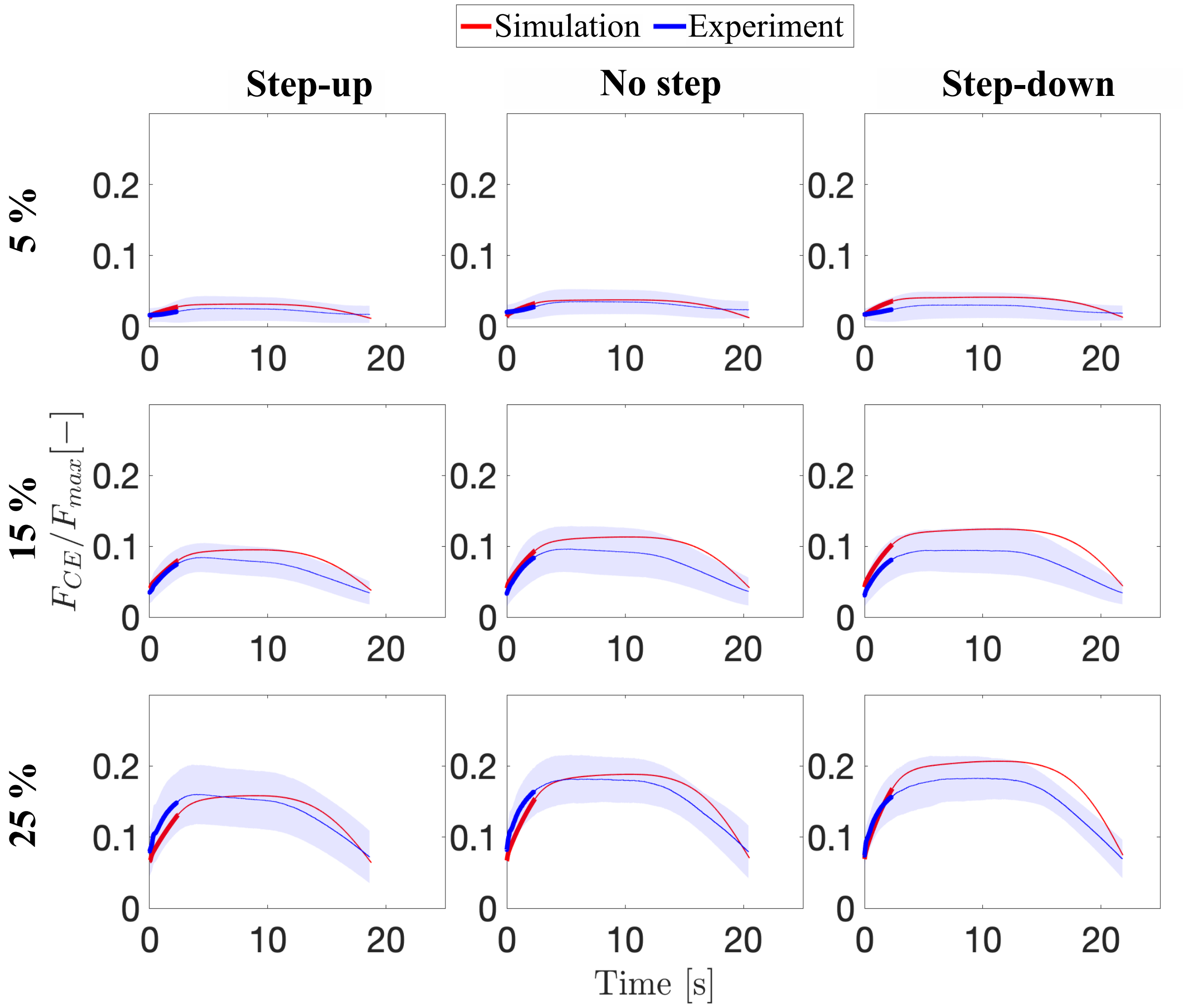

Figure S7. Force generated in quasistatic-scenario by both muscle fibers and the Hill-type muscle model during one hopping cycle — from touch-down to toe-off — are presented for all perturbation and activity levels. The thick and thin sections of the loops represent the preflex and remaining part of the stretch-shortening cycle, respectively. the blue line and shaded area represent the mean of all experimental trials and the standard deviation, respectively.

Table S2. Statistical comparison of muscle fibers' perturbation response for each activity level. Significantly different results are indicated by *; *a*: activity level; SRS: short range stiffness; χ^2: test value of Friedmantest; *p*: significance value; *r*: effect size; P0: no-perturbation; P↑: step-up perturbation; P↓: step-down perturbation

Condition	*a*	Parameter	χ^2	*p*	P0 vs. P↑(*r*)	P0 vs. P↓(*r*)	P↓ vs. P↑ (*r*)
Dynamic-Scenario	5 %	Preflex work	8.222	0.016*	0.029(.41)*	1	0.055
		SRS	0.889	0.641	-	-	-
		Work Fig. 5A	1.556	0.459	-	-	-
	15 %	Preflex work	16.222	0.001*	0.055	0.297	0.001(0.63)*
		SRS	0	1	-	-	-
		Work Fig. 5A	4.667	0.097	-	-	-
	25 %	Preflex work	14.889	0.001*	0.029(0.41)*	0.716	0.001(0.59)*
		SRS	1.556	0.459	-	-	-
		Work Fig. 5A	3.556	0.169	-	-	-
Quasistatic-Scenario	5 %	Preflex work	4.222	0.121	-	-	-
		Stiffness	4.222	0.121	-	-	-
	15 %	Preflex work	16.222	0.001*	0.055	0.297	0.001(0.63)*
		Stiffness	0.667	0.717	-	-	-
	25 %	Preflex work	16.222	0.001*	0.055	0.297	0.001(0.63)*
		Stiffness	0.667	0.717	-	-	-
Dynamic vs. Quasistatic-Scenario Fig. 5B	5 %	Preflex work	2.667	0.264	-	-	-
	15 %	Preflex work	4.222	0.121	-	-	-
	25 %	Preflex Work	1.556	0.459	-	-	-

Table S3. Statistical comparison of activity differences for each perturbation case. Significantly different results are indicated by *; effect size is shown for significant differences in (); SRS: short range stiffnes; χ^2: test value of Friedmantest; p: significance value; P0: no-perturbation; P↑: step-up perturbation; P↓: step-down perturbation

Condition	P	Parameter	χ^2	p	5 % vs. 15 %	5 % vs. 25 %	15 % vs. 25 %
Dynamic-Scenario	P0	Preflex work	18	0.001*	0.102	0.001(0, 67)*	0.102
		SRS	18	0.001*	0.102	0.001(0.67)*	0.102
		Work Fig. 5A	12.667	0.002*	0.472	0.001(0.56)*	0.102
	P↑	Preflex work	18	0.001*	0.102	0.001(0.67)*	0.102
		SRS	18	0.001*	0.102	0.001(0.67)*	0.102
		Work Fig. 5A	18	0.001*	0.102	0.001(0.67)*	0.102
	P↓	Preflex work	18	0.001*	0.102	0.001(0.67)*	0.102
		SRS	18	0.001*	0.102	0.001(0.67)*	0.102
		Work Fig. 5A	18	0.001*	0.102	0.001(0.67)*	0.102
Quasistatic-Scenario	P0	Preflex work	16.22	0.001*	0.055	0.001(0.63)*	0.297
		Stiffness	12.667	0.002*	0.102	0.001(0.56)*	0.472
	P↑	Preflex work	16.22	0.001*	0.055	0.001(0.63)*	0.297
		Stiffness	11.556	0.003*	0.055	0.003(0.52)*	1
	P↓	Preflex work	18	0.001*	0.102	0.001(0.67)*	0.102
		Stiffness	11.556	0.003*	0.055	0.003(0.52)*	1
Dynamics vs. Quasistatic-Scenario Fig. 5B	P0	Preflex work	2	0.368	-	-	-
	P↑	Preflex work	0.222	0.895	-	-	-
	P↓	Preflex work	0.222	0.895	-	-	-

Table S4. Statistical comparison between Dynamic and Quasistatic Scenario. Significantly different results are indicated by *; SRS: short range stiffnes; *z*: test value of t-test; *p*: significance value; *r*: effect size; P0: no-perturbation; P↑: step-up perturbation; P↓: step-down perturbation

Activity Level	Parameter	P	z	p	r
5 %	Preflex work	P0	0.533	0.594	-
		P↑	0.296	0.767	-
		P↓	1.333	0.182	-
	SRS	P0	0	1	-
		P↑	0	1	-
		P↓	0	1	-
15 %	Preflex work	P0	0.667	0.505	-
		P↑	0.533	0.594	-
		P↓	0.667	0.505	-
	SRS	P0	1.333	0.182	-
		P↑	2	0.046*	0.67
		P↓	2.666	0.008*	0.89
25 %	Preflex work	P0	0.667	0.505	-
		P↑	0.652	0.515	
		P↓	0.667	0.505	-
	SRS	P0	2.666	0.008*	0.89
		P↑	2.666	0.008*	0.89
		P↓	2	0.046*	0.67
